# Association Between Intergenerational Support, Technology Perception and Trust, and Intention to Seek Medical Care on the Internet Among Chinese Older Adults: Cross-Sectional Questionnaire Study

**DOI:** 10.2196/65065

**Published:** 2025-01-06

**Authors:** Hengjiang Jin, Ying Qu

**Affiliations:** 1 School of Journalism Chongqing University Chongqing China

**Keywords:** intergenerational support, older adults, internet medical intentions, perceived technology, trust

## Abstract

**Background:**

Avoiding technological innovation does not simplify life. In fact, using internet-based medical services can enhance the quality of life for older adults. In the context of an aging population and the growing integration of information technology, the demand for internet-based medical services among older adults is gaining increased attention. While scholars have highlighted the important role of intergenerational support in promoting digital inclusion for older adults, research on the relationship between intergenerational support and older adults’ intentions to seek online care remains limited.

**Objective:**

This study aims (1) to explore the association between intergenerational support, online medical information, and older adults’ intention to seek medical care online, and (2) to examine the mediating role of technology perception and trust, as well as the moderating role of eHealth literacy.

**Methods:**

A cross-sectional survey was conducted in China, collecting 958 valid responses from older adults aged 60 years and above. A vast majority of participants were between the ages of 60 and 75 years (771/958, 80.5%). Of the 958 participants, 559 (58.4%) resided in rural areas, while 399 (41.6%) lived in urban areas. The survey included questions on intergenerational support, perceived usefulness, perceived ease of use, trust, online medical information, eHealth literacy, and the intention to seek medical care online. Structural equation modeling and linear regression analysis were applied to explore the relationship between intergenerational support and the intention to seek medical care on the internet.

**Results:**

Intergenerational support was positively associated with perceived ease of use (β=.292, *P*<.001), perceived usefulness (β=.437, *P*<.001), trust (β=.322, *P*<.001), and the intention to seek medical care online (β=.354, *P*<.001). Online medical information also positively affected the intention to seek medical care online among older adults (β=.109, *P*<.001). Perceived ease of use (β=.029, 95% CI 0.009-0.054), perceived usefulness (β=.089, 95% CI 0.050-0.130), and trust (β=.063, 95% CI 0.036-0.099) partially mediated the association between intergenerational support and the intention to seek medical care online. Further analysis found that perceived ease of use, perceived usefulness, and trust played a chain mediating role between intergenerational support and the intention to seek medical care online (β=.015, 95% CI 0.008-0.027; β=.022, 95% CI 0.012-0.036). Additionally, eHealth literacy played a moderating role in the relationship between intergenerational support and perceived ease of use (β=.177, *P*<.001), perceived usefulness (β=.073, *P*<.05), trust (β=.090, *P*<.01), and the intention to seek medical care online (β=.124, *P*<.001).

**Conclusions:**

An integrated model of health communication effects was constructed and validated, providing empirical support for the intention to seek medical care online and for the impact of health communication. This model also helps promote the role of technology in empowering the lives of seniors.

## Introduction

### Background

The internet has become an important medium for providing various eHealth services and a source of medical information [1]. Smartphones bring the convenience of telemedicine to users’ fingertips [2]. According to the 52nd China Internet Network Information Center report [3], China has 364 million internet medical care users, accounting for 33.8% of all internet users. The number of online hospitals continues to grow, and the development of large-scale online medical care platforms shows strong momentum.

The World Health Organization has encouraged member states to actively use digital technologies to improve medical, health, and social services, thereby promoting active aging [4]. Given that older adults are the primary users of health care services [5], there is significant potential for internet-based medical services to enhance geriatric health. Currently, China’s population is aging rapidly [6]. As of the end of 2023, the population of older adults in China (aged ≥60 years) has reached 297 million, accounting for 21.1% of the total population. For older adults, proficient use of technology is key to accessing internet-based medical services [7]. However, digital disparities—such as difficulties in acquiring, operating, and using basic equipment, as well as insufficient knowledge—limit their ability to equally benefit from technological advancements. Given the trend of population aging, the challenge of successfully integrating older adults into the digital health ecosystem has become a pressing issue.

Some prior studies have explored this question, noting that older adults are particularly concerned about the quality and risks of internet-based medical services [8]. Additionally, older adults’ habits play an important role in explaining their use of such services [9]. However, further discussion is needed to clarify the complexities and mechanisms that influence older adults’ intention to seek medical care online.

### Seeking Medical Care Online

Intention to adopt internet-based medical services refers to individuals’ subjective beliefs and willingness to seek care through such services when facing health problems [10]. It reflects the importance individuals place on their health, as well as their trust in and reliance on internet-based medical services. This intention is influenced by multiple factors, including disease severity, individual perceptions and attitudes toward the illness, and the quality and availability of health care services.

Examinations of the intention to adopt internet-based medical services currently follow 3 approaches. The first approach is based on the Technology Acceptance Model or the Unified Theory of Acceptance and Use of Technology [11], which assesses user acceptance of online health platforms and the willingness of different population groups to use internet-based medical services [12]. The second approach constructs an analytical model centered on trust within the doctor-patient relationship, focusing on factors such as personal trust preferences and website trustworthiness [13]. The third approach is based on Social Exchange Theory, which examines the physicians’ perspectives on the potential impact of interactive tools on their careers [14], as well as how regulatory systems, reputation systems, and communication exchanges shape their motivation to use these services [15].

Examining older adults’ access to online health care in greater detail, Mansson et al [16] argued that the use of mobile health (mHealth) apps may reduce treatment costs. However, Askari et al [17] identified several factors influencing older adults’ willingness to use mHealth apps, including perceived usefulness (PU), ease of use, social connections, social norms, and anxiety.

### Intergenerational Support

Intergenerational Support Theory asserts that the flow of resources among family members is bidirectional [18], encompassing economic, technological, and emotional exchanges [19]. In the context of internet-based medical services, intergenerational support focuses on the technical assistance provided by the children to their parents in using these services.

Perceived ease of use (PEOU) measures older adults’ ratings of technology’s friendliness versus their beliefs about ease of mastery [20]. However, many older adults are unfamiliar with smartphone operation, are often excluded from various internet services, and are unable to benefit equally from digital technologies [21]. Additionally, they perceive apps as complex [22], resulting in low overall levels of PEOU. The lack of digital literacy and the challenges of aging are significant barriers to older adults’ intention to use mHealth technology [23]. In the context of online-based medical services, children can assist older adults by guiding them through the process of using online platforms—such as downloading, registering, finding, and consulting internet-based medical services. They can also help resolve technical issues, thereby reducing frustration [24]. This support will make it easier for older adults to use these services, leading to an increase in their assessment of PEOU. Based on this, the following hypotheses are proposed.

- Hypothesis 1a: Intergenerational support is positively associated with perceived ease of use of internet-based medical services among older adults.

PU reflects older people’s views that a particular technology can improve their health care experience. It encompasses their belief that internet-based medical services can meet their health management needs, such as online appointment booking, teleconsultation, and e-prescription flow. Studies have shown that older adults are more likely to adopt technology when they gain insight into its usefulness and potential benefits. Through direct operation or demonstration by children, older adults can independently or semi-independently use the internet-based medical service platform to experience its advantages in simplifying clinical services, saving time and energy for medical treatment, and enhancing their knowledge and acceptance of the platform. Once older adults experience the tangible benefits of internet-based medical services, it may stimulate positive feedback mechanisms that gradually ease the psychological barriers to adopting new technologies. This, in turn, can enhance psychological adaptation and cognitive health [25], thereby deepening the PU of the internet-based medical service platform.

- Hypothesis 1b: Intergenerational support is positively associated with the perceived usefulness of internet-based medical services for older adults.

Trust typically builds up gradually through continuous and intimate interactions, which can enhance patients’ positive expectations [26]. However, many older adults have conservative and negative attitudes toward new technologies and are hesitant to use online services. As a result, it can be challenging for services such as internet-based medical services to gain the trust of older adults [27]. According to Ma et al [28], trust serves as a critical criterion for older adults in identifying individuals who can help them enhance their digital skills. Consequently, the younger generation plays a significant role in building trust and security for older adults. This support also involves a sense of being recognized and respected [29]. We hypothesize that greater intergenerational support is more likely to reduce older adults’ unfamiliarity and thus increase their trust in these services.

- Hypothesis 1c: Intergenerational support is positively associated with older adults’ trust in internet-based medical service.

According to Li and Kostka [30], social support plays a significant role in affecting older adults’ digital engagement. Many older adults who maintain close contact with their families tend to receive more effective support for digital learning and engagement [30]. Additionally, given the importance of filial piety in Chinese culture, adult children are expected to assist disadvantaged family members [31]. This includes providing help and counseling regarding digital access, use, and literacy, as well as facilitating bottom-up technology transfer [32] to help their parents accomplish reverse socialization [33]. As a result, support and feedback can effectively enhance the digital competence of older adults [34] and promote the adoption of new technologies.

- Hypothesis 1d: Intergenerational support is positively associated with older adults’ intention to seek medical care on the internet.

### Perceived Usefulness and Perceived Ease of Use

As the key elements determining users’ behavioral intentions, PU indicates the extent to which using a particular system or operation can improve task performance or achieve a specific goal. PEOU reflects the user’s subjective evaluation of the convenience and ease provided by using something [35].

Some studies have shown that the PU of social media affects people’s trust in the platform as well as the channel [36]. Acharya et al [37] further found that the PU of recommender systems can directly impact consumers’ trust. It has also been confirmed that PEOU is positively correlated with consumer trust [38]. This study suggests, therefore, that older adults may trust internet-based medical services if they perceive them as useful or easy to use.

- Hypothesis 2a: Perceived ease of use is positively associated with older adults’ trust in internet-based medical service.
- Hypothesis 2b: Perceived usefulness is positively associated with older adults’ trust in internet-based medical service.

Currently, technology perception has been studied in various contexts, such as the adoption of smart home technologies [39] and the willingness to use internet applications [40]. Naidoo and Leonard [41] found that the continued willingness to use e-services is entirely dependent on high levels of PU. Further research indicates that the PU of the internet is an important predictor of an individual’s use of eHealth solutions and plays a significant role in patients’ sustained use of online health care communities [42]. For older adults, the functions of internet-based medical services are far more complex than simple services such as browsing short videos. Older patients tend to consider using these services only if the transition to new medical treatments is easy [43]. This study therefore argues that older adults are more likely to use internet-based medical services if they perceive them to be useful or easy to use.

- Hypothesis 3a: Perceived ease of use is positively associated with older adults’ intention to seek medical care on the internet.
- Hypothesis 3b: Perceived usefulness is positively associated with older adults’ intention to seek medical care on the internet.

### Trust

Schoorman et al [44] suggested that trust in internet-based medical services is the tendency for people to believe that these services can fulfill their health needs and to be willing to use them, reflecting the level of acceptance for such services [45]. Trust is a crucial factor for assessing users’ willingness to use internet-based medical services [46], and establishing trust improves the likelihood of older adults using these services [47]. This study therefore suggests that older adults who trust internet-based medical services are more likely to be willing to access such services. We therefore propose the following hypothesis:

- Hypothesis 4: Trust is positively associated with older adults’ intention to seek medical care on the internet.

### Online Medical Information

Online medical information—encompassing all types of medical-related content delivered and shared through internet platforms such as search engines, medical websites, and online forums—is highly sought after. The causes and treatments of diseases are among the most popular topics for medical information searches [48]. Notably, 60% of individuals view online medical information as equally good or better than information provided by physicians [49]. Online medical information enhances the efficiency of medical knowledge dissemination and is rapidly replacing traditional approaches for seeking advice and information [50]. It helps to improve individuals’ knowledge, promote disease prevention, and facilitate access to appropriate medical services. To pursue more informed health decisions [51], older adults often seek health-related medical information online, which influences their health behaviors [52]. Consequently, older adults may be motivated to seek guidance from physicians practicing online [53]. The following research hypothesis is therefore proposed:

- Hypothesis 5: Online medical information is positively associated with older adults’ intention to seek internet-based medical services.

### eHealth Literacy

eHealth literacy refers to the ability needed to access, understand, and evaluate health information from digital resources and make informed health decisions [54]. It encompasses active information-seeking, 2-way interactive communication, and information utilization/sharing [55]. Although older adults are increasingly relying on the internet to access health-related services, they often struggle to meet the necessary eHealth literacy requirements [56]. Families play an important role in promoting eHealth literacy among older adults [57]. By strengthening intergenerational support, they can help older adults better learn and apply new technologies. When confronted with a complex internet health care delivery system, families can assist in quickly getting up to speed and becoming proficient in its operation. At this level, internet health care services are easy to use. At the same time, families can help older adults obtain rich health information from the platform [58] and manage their daily health needs, which can enhance their perception of the usefulness of these services. Therefore, we propose the following:

- Hypothesis 6a: eHealth literacy positively moderates the relationship between intergenerational support and perceived ease of use.
- Hypothesis 6b: eHealth literacy positively moderates the relationship between intergenerational support and perceived usefulness.

A study found that social interaction, access to technology, and digital literacy are positively correlated. With the assistance of their children, older adults can significantly improve their ability to access, understand, and apply digital health information [59]. High levels of eHealth literacy can increase older adults’ confidence and ability to assess the reliability and validity of internet-based health care services, thereby enhancing their trust in such services.

- Hypothesis 6c: eHealth literacy positively moderates the relationship between intergenerational support and trust.

According to Health Empowerment Theory, eHealth literacy and social support can promote self-care behaviors among older adults [60]. The findings of Hsu et al [61] further indicate that individuals with higher levels of eHealth literacy have a greater potential to make informed health choices, improved health care competence, and ultimately higher quality of life. Therefore, we propose the following:

- Hypothesis 6d: eHealth literacy positively moderates the relationship between intergenerational support and intention to seek medical care on the internet.

## Methods

### Data Collection and Participants

In this study, a questionnaire (Multimedia Appendix 1) was used to validate the research model. To ensure reliability and validity, all items were borrowed from well-established domestic and international scales and adjusted according to the theme of internet medical care. The questionnaire comprises a total of 7 latent variables, involving 24 items, all measured on a 7-point Likert scale. Before the official survey, a presurvey was conducted with 50 older adults to ensure a high degree of internal consistency. Based on their feedback, questions with ambiguities or semantic problems were adjusted.

Between March 20, 2024, and March 31, 2024, we officially launched the distribution of the questionnaire using a combination of online and offline methods. This approach ensures that the data (Multimedia Appendix 2) collected are broad and representative, providing a more comprehensive understanding of the attitudes and needs of older adults regarding the use of the internet for health care services. The study was conducted among individuals aged 60 years and older who had autonomous behavioral skills and some experience with internet use, encompassing both rural and urban areas. Those who lacked basic knowledge of the internet or were unable to participate due to daily activities or cognitive limitations were excluded.

Online questionnaires offer the advantages of low cost, autonomy, and comprehensive documentation, as well as the ability to effectively expand the range of respondents. In this study, an online self-report questionnaire was designed using Questionnaire Star (Changsha Ranxing Information Technology Co., Ltd.). The questionnaire was distributed through a convenience sample with a snowball approach. An online survey link was shared across various social media platforms and WeChat (Tencent Holdings Limited) app groups, where participation was voluntary. This approach covered older adult groups from regions such as Chongqing, Hunan, Guizhou, and Zhejiang, among others. To address the unfamiliarity of some older adults with the operation of the Questionnaire Star and the limitation of their text reading comprehension ability, this study required them to complete the questionnaire in the presence and under the guidance of their middle-aged or young relatives or friends. This approach ensured the accuracy and reliability of the data by providing assistance with understanding and navigating the questions.

Although many older people already have smart devices, they may be wary of clicking on a link to participate in an online questionnaire. Therefore, face-to-face interviews are particularly necessary. In the offline questionnaire survey, we adopted a dual strategy: on the one hand, the research team personally visited communities and villages in Chongqing and Hunan, directly communicating with older adult groups through on-site visits to ask them in detail about their willingness to use internet health care services. On the other hand, with the support of governmental agencies where the researchers are domiciled, we commissioned local staff to assist in distributing some of the questionnaires. For the older adults interviewed who had the ability to write, we asked them to fill in the questionnaire themselves; for patients who were unable to fill in the questionnaire by themselves, their primary caregiver or interviewer filled in the questionnaire on their behalf. The questionnaires were collected on-site after completion.

During the data collection phase, a total of 1200 questionnaires were collected. After eliminating invalid questionnaires (including online questionnaires that took less <90 seconds to complete, failed the honesty test, had missing values, or provided the same answer to all questions, as well as offline questionnaires that were obviously invalid, such as choosing “strongly agree” for all of them), a total of 958 valid questionnaires were ultimately obtained for an effective response rate of 79.8%.

### Measurements

#### Intergenerational Support

The measurement items for this dimension were derived from Lang and Schütze’s study [62] and consisted of 3 items. It was measured with good reliability (α=.887) [63]. This factor collectively explained 81.6% of the variance (Kaiser-Meier-Olkin [KMO]=0.748). Specific items included “My children encourage me to seek medical care on the internet, and I am willing to try it,” “My children guide I would be willing to try,” and “When I have problems with internet health care, my children help me solve the problem, so I would be willing to try it.”

#### Online Medical Information

Based on a study by de Boer et al [64], 4 items were used to measure the medical information that older adults sought online. The measurement items included “I would search for medical information on the internet” and “Online medical information has taught me something about health.” The results of factor analysis for the 4 items showed a KMO value of 0.843, with the factor explaining a total of 76.2% of the variance; these items also had good reliability (α=.895).

#### Trust

This dimension is based on the studies by Fogel and Nehmad [65] and Deng et al [66] and consists of 3 items. Specific items included “Most of the doctors on the online medical service platforms are health experts in their fields, and I have no doubt about their professionalism” and “Generally, I trust the health advice or tips from doctors on the internet.” The overall performance of these items indicated good reliability (α=.868); this factor collectively explained 79.2% of the variance (KMO=0.735).

#### Perceived Ease of Use

The 5 items were based on the study by Deng et al [66]. Typical items include “I don’t think it is difficult to use the internet for health counseling” or “In general, I think internet health care is easy to use.” The results of factor analysis for the 3 items showed a KMO value of 0.885, explaining a total of 72.7% of the variance. The 5 items showed good reliability (α=.906).

#### Perceived Usefulness

The items for this factor were based on those in the study by Deng et al [66]. The items included “I believe that using the internet for medical care can improve the efficiency of health care” and “Overall, I think online medical care is useful for health management.” The results of the analysis showed a KMO value of 0.746, explaining a total of 81.7% of the variance, and good reliability (α=.888).

#### Intention to Seek Medical Care on the Internet

These 3 items referred to the study by Deng et al [66]., including “When I face health problems, I think I will solve them through internet health care” and “I am willing to use internet health care services for health counseling, such as disease control.” These analyses showed a KMO value of 0.751, explaining a total of 82.8% of the variance; all 3 items also had good reliability (α=.895).

#### eHealth Literacy

This dimension combines health and media literacy and references the study by Norman and Skinner [67]. The 3 items included “I know how to find useful health resources and messages online” or “I know how to use the health care-type information I find on the internet to help myself,” with 82.6% variance explained, a KMO value of 0.751, and good reliability (α=.894).

All 24 items were rated on a 7-point Likert scale (1=strongly disagree, and 7=strongly agree). All measurement items can be found in Table S1 in Multimedia Appendix 3.

### Data Analysis

Cronbach α was used to determine the reliability of the scales. Construct validity and reliability were examined using exploratory factor analysis with varimax rotation, Bartlett test, and KMO statistics, as well as confirmatory factor analysis (CFA).

For the analysis of structural equation modeling, Amos 26.0 (IBM Corp.) was used to analyze the data and the effect of the model was measured by the fit indices, which included chi-square/degree of freedom (*χ*^2^/*df*), the goodness of fit index (GFI), the adjusted goodness of fit index (AGFI), the incremental fit index (IFI), the comparative fit index (CFI), the nonnormed fit index (NNFI), and root mean square error of approximation (RMSEA). Additionally, the bootstrap method was used to test the mediation model with 5000 iterations of repeated sampling. Results were also obtained using SPSS 26.0 (IBM Corp.) to test for interaction effects.

### Ethical Considerations

Ethical approval for this study was obtained from the academic committee (acting as the ethics committee) of the School of Journalism and Communication at Chongqing University (approval number 20240320). Informed consent was obtained from all participants before their participation in the survey. Participants were provided with a detailed informed consent form outlining the purpose of the study, the duration of the investigation, the procedures, and the potential risks and benefits. They were informed that their participation was voluntary and that they had the right to withdraw at any time without facing any negative consequences. Participants were assured of the confidentiality and anonymity of their responses.

## Results

### Participant Characteristics

The sample demographics are as follows: of the 958 participants, 771 (80.5%) were between the ages of 60 and 75 years, 511 (53.3%) were females, and 447 (46.7%) were males. In terms of residential area, 559 (58.4%) participants resided in a rural area and 399 (41.6%) in an urban area. Most participants (812/958, 84.8%) had between 1 and 3 children, about one-quarter (214/958, 22.3%) of the participants reported an education level of high school and above, nearly half (440/958, 45.9%) rated their self-assessed health status as fair, and 186 (19.4%) rated it as poor or very poor. Detailed demographic information is presented in Table 1.

**Table 1:** Participant characteristics (N=958).

Characteristics			Values, n (%)
**Age (years)**			
	60-65	444 (46.3)	
	66-70	183 (19.1)	
	71-75	144 (15.0)	
	76-80	123 (12.8)	
	>80	64 (6.7)	
**Gender**			
	Women	511 (53.3)	
	Male	447 (46.7)	
**Status of residence**			
	Residence with spouse	424 (44.3)	
	Large family living together	188 (19.6)	
	Living with children	184 (19.2)	
	Living alone	137 (14.3)	
	Others	25 (2.6)	
**Educational level**			
	Secondary schools	353 (36.8)	
	Junior high school	243 (25.4)	
	Not attending school	148 (15.4)	
	High school/secondary school	138 (14.4)	
	Undergraduate	53 (5.5)	
	Graduate students and above	23 (2.4)	
**Occupation now or before retirement**			
	Peasants	475 (49.6)	
	Workers	112 (11.7)	
	Others	109 (11.4)	
	Public officials/units	107 (11.2)	
	Sole trader/freelancer	100 (10.4)	
	Business managers/office staff	55 (5.7)	
**Monthly income (CNY^a^)**			
	≤2000	472 (49.3)	
	2001-4000	286 (29.9)	
	4001-6000	133 (13.9)	
	6001-8000	44 (4.6)	
	≥8001	23 (2.4)	
**Urban/rural**			
	Countryside	559 (58.4)	
	Cities and towns	399 (41.6)	
**Number of children**			
	2	452 (47.2)	
	3	192 (20.0)	
	1	168 (17.5)	
	4	87 (9.1)	
	5	30 (3.1)	
	0	18 (1.9)	
	≥6	11 (1.1)	
**Self-assessed health status**			
	General	440 (45.9)	
	Better	288 (30.1)	
	Rather poor	170 (17.7)	
	Rare	44 (4.6)	
	Very poor	16 (1.7)	

^a^1 CNY=US $0.14.

### Structural Model

The CFA model was fit to the data (Table S2 in Multimedia Appendix 3): *χ*^2^_231_=525.021, GFI=0.958, AGFI=0.945, IFI=0.980, CFI=0.980, NNFI=0.976, and RMSEA=0.036. The model fit appeared to be good, and all items met the criteria proposed by Hu and Bentler [68] and Kline [69].

According to Bagozzi and Youjae [70] and Hair [71], composite reliability (ρ)≥0.60 and average variance extracted ≥0.50 indicate good internal consistency and convergent validity. We therefore used composite reliability to measure the reliability of the constructs, with the following results: intergenerational support=0.887, online medical information=0.896, trust=0.869, PU=0.888, PEOU=0.906, eHealth literacy=0.894, and intention to seek medical care on the internet=0.896. As shown in Table S3 in Multimedia Appendix 3, the factor loadings for all question items were greater than 0.60. Convergent validity was assessed using the average variance extracted, which yielded the following values: 0.723 for intergenerational support, 0.684 for online medical information, 0.690 for trust, 0.726 for PU, 0.659 for PEOU, 0.739 for eHealth literacy, and 0.742 for intention to seek medical care on the internet. These results indicate good convergent validity. As shown in Table S4 in Multimedia Appendix 3, the square root of the average variance extracted for each construct is greater than the correlation coefficients between the variables, indicating good discriminant validity [72].

Based on the final CFA model, a full structural equation modeling was conducted to test the hypotheses. The model fit indices (Table S5 in Multimedia Appendix 3) were as follows: *χ*^2^_179_=498.30, GFI=0.954, AGFI=0.940, IFI=0.975, CFI=0.975, Tucker-Lewis Index=0.971, and RMSEA=0.043. These indices indicate a good model fit, and the structural equation modeling approach allowed the constructed and hypothesized model to be tested in a more satisfactory manner.

### Association of Intergenerational Support and Other Variables and Intention to Seek Medical Care on the Internet

Table 2 shows the results of the path analysis. Intergenerational support was positively associated with PEOU, PU, trust, and older adults’ intention to seek medical care on the internet (β=.292, *P*<.001; β=.437, *P*<.001; β=.322, *P*<.001; and β=.354, *P*<.001, respectively); therefore, hypotheses H1a, H1b, H1c, and H1d were supported. PEOU and PU were positively associated with older adults’ trust in internet-based medical services (β=.263, *P*<.001 and β=.261, *P*<.001, respectively), supporting H2a and H2b. PEOU and PU were also positively correlated with older adults’ intention to seek medical care on the internet (β=.099, *P*=.002 and β=.204, *P*<.001, respectively), supporting H3a and H3b. Trust was positively correlated with older adults’ intention to seek medical care on the internet (β=.197, *P*<.001), supporting H4. Online medical information was also positively correlated with older adults’ intention to seek medical care on the internet (β=.109, *P*<.001), supporting H5.

**Table 2:** Results for direct relationships.

Relationship	Path coefficient	SE	Critical ratio	*P* value
PEOU ← IS^a^	0.292	0.037	8.204	<.001
PU ← IS	0.437	0.035	12.388	<.001
T ← IS	0.322	0.034	8.543	<.001
T ← PEOU^b^	0.263	0.029	8.003	<.001
T ← PU^c^	0.261	0.032	7.345	<.001
SMCI^d^ ← IS	0.354	0.04	9.265	<.001
SMCI ← T^e^	0.197	0.046	4.961	<.001
SMCI ← PEOU	0.099	0.032	3.088	.002
SMCI ← PU	0.204	0.037	5.809	<.001
SMCI ← OMI^f^	0.109	0.034	3.781	<.001

^a^IS: intergenerational support.

^b^PEOU: perceived ease of use.

^c^PU: perceived usefulness.

^d^SMCI: intention to seek medical care on the internet

^e^T: Trust

^f^OMI: online medical information.

Table 3 shows the mediating relationship between PU, PEOU, and trust. The total effect value of the relationship between intergenerational support and willingness to use internet-based medical services was 0.573, while the direct effect value was 0.354. Both had a positive 95% CI and a *P* value of less than 0.05, indicating that the total and direct effects were significant. The indirect effect of trust was 0.063 (95% CI 0.036-0.099). More specifically, trust partially mediated the association between intergenerational support and older adults’ willingness to use internet-based medical services. PEOU, PU, and trust also played a chain mediating role between intergenerational support and willingness to use internet-based medical services, with specific mediation effect sizes of 0.015 and 0.022, respectively. The 95% CI for the PEOU path was 0.008-0.027, and for the PU path, it was 0.012-0.036. The *P* values for both chain mediation paths were less than 0.05, suggesting that intergenerational support can enhance PEOU and PU among older adults. This, in turn, enhances trust, which subsequently increases the willingness to use internet-based medical services.

**Table 3:** Total, direct, and indirect effects.^a^

Mediation effect and path			Estimate		95% CI		*P* value
**Total effect**							
	IS^b^ → SMCI^c^	0.573		0.509-0.632		<.001	
**Direct effect**							
	IS → SMCI	0.354		0.278-0.428		<.001	
**Indirect** **effect**							
	IS → T^d^ → SMCI	0.063		0.036-0.099		<.001	
	IS → PEOU^e^ → SMCI	0.029		0.009-0.054		0.005	
	IS → PU^f^ → SMCI	0.089		0.050-0.130		<.001	
	IS → PEOU → T → SMCI	0.015		0.008-0.027		<.001	
	IS → PU → T → SMCI	0.022		0.012-0.036		<.001	

^a^Standardized estimation of 5000 bootstrap samples.

^b^IS: intergenerational support.

^c^SMCI: intention to seek medical care on the internet.

^d^T: trust.

^e^PEOU: perceived ease of use.

^f^PU: perceived usefulness.

**Figure 1:**
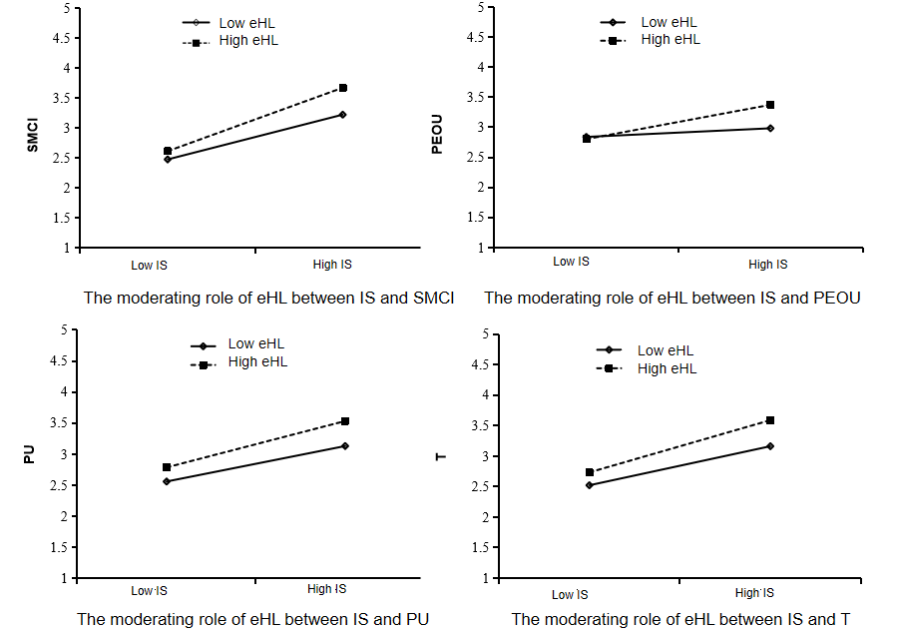
Simple slope plot. eHL: eHealth literacy; IS: intergenerational support; PEOU: perceived ease of use; PU: perceived usefulness; SMCI: intention to seek medical care on the internet; T: trust.

Table 4 shows the moderating relationships of eHealth literacy. The interaction term between intergenerational support and eHealth literacy was a significant positive predictor of PEOU (β=.177, *P*<.001) and PU (β=.073, *P*=.018). This suggests that eHealth literacy moderates the effect of intergenerational support on PEOU and PU, thereby supporting H6a and H6b. The interaction term between intergenerational support and eHealth literacy was a significant positive predictor of trust (β=.09, *P*<.01), suggesting that eHealth literacy moderates the effect of intergenerational support on trust, thus supporting H6c. The interaction term between intergenerational support and eHealth literacy had a significant positive predictive effect on the intention to seek medical care on the internet (β=.124, *P*<.001). This indicates that eHealth literacy moderates the influence of intergenerational support on the intention to seek medical care on the internet, thereby supporting H6d. A further simple slope analysis was then conducted (Figure 1). The slope was shallower when eHealth literacy was low (solid line) and steeper when eHealth literacy was high (dashed line). This suggests that intergenerational support was a more significant positive predictor of the intention to seek medical care on the internet, PEOU, PU, and trust at high levels of eHealth literacy.

**Table 4:** Moderated effect.

Path	Intention to seek medical care on the internet, β (*P* value)	Perceived ease of use, β (*P* value)	Perceived usefulness, β (*P* value)	Trust, β (*P* value)
Intergenerational support	.447 (<.001)	.183 (<.001)	.337 (<.001)	.389 (<.001)
Electronic health literacy^a^	.15 (<.001)	.094 (.002)	.167 (<.001)	.173 (<.001)
IS^b^ × eHL^c^	.124 (<.001)	.177 (<.001)	.073 (.018)	.09 (.003)

^a^Electronic health literacy was used as a moderating variable in the model after controlling for the variables age, gender, education, residential status, occupation now or before retirement, monthly income, urban /rural, number of children, self-assessed health status.

^b^IS: intergenerational support.

^c^eHL: eHealth literacy.

## Discussion

### Principal Findings

Older people are often referred to as digital refugees [73]. A lack of digital skills may contribute to the risk of widening health disparities and unequal access to services [74]. Studies have shown that many older individuals face numerous barriers to using smartphones, often due to a lack of knowledge about apps and their features [75] and technology anxiety [76]. Most older adult patients are also accustomed to face-to-face outpatient services, making the adoption of internet-based health care not easily achievable for this population. Although the digital divide hinders active aging by reducing the social participation, as well as the physical and mental health, of older adults [77], the active bridging provided by intergenerational support helps older adults overcome their fears, improve their digital skills, and enhance their sense of well-being and accessibility. This, in turn, facilitates their integration into the digital age by fostering a positive and optimistic mindset that promotes effective active aging [78].

Intergenerational support to address the technical aspect occurs when children explain to older adults how to operate the online medical platform, help them understand error messages, and provide tips on various operations such as registration, logging in, booking appointments, and online counseling [79]. This support involves not only technical guidance but also spiritual care and comfort. This kind of benign emotional exchange can promote older people’s perception of satisfaction, enhancing their acceptance of new technologies. Therefore, online medical care with intergenerational support is not only a reflection of filial piety but also an endorsement of positive intergenerational relationships. Children’s counseling and assistance can improve the cognitive abilities [80] and overall health awareness of older adults, alleviate their fear of technology, reduce digital loneliness, and strengthen their confidence in using internet-based medical services.

The results of this study show that the higher the level of intergenerational support, the greater the PEOU and usefulness of internet health care platforms among older adults, which in turn enhances their willingness to access these services. Through the help and guidance of family members, older adults are able to master internet technologies more quickly and effectively utilize these tools for health management. This support not only increased older adults’ PEOU of internet-based health care services but also boosted their confidence and security, leading to a greater willingness to try and use these services. In addition, this support implies encouragement and companionship on an emotional and psychological level [81]. When older adults feel the concern and attention from their family members or later generations regarding their health problems, and the possibility of obtaining a convenient and professional health care experience through internet health care services, they are more likely to develop positive psychological expectations that these services are valuable and can practically solve their health issues [82]. This positive cognitive shift directly enhances older adults’ acceptance of and reliance on internet health care services, thereby strengthening their willingness to seek medical care.

There is also a transformational path between social support factors that generate the intention to adopt internet-based medical services; trust mediates between intergenerational support and the intention to adopt internet-based medical services. Trust, in this context, refers to the extent to which an individual relies on an internet-based medical service to meet health management needs [83], encompassing aspects such as the professionalism of the health care service, platform security, and a positive user experience. Cao et al [84] suggested that mistrust is a common reason for digital disengagement and that trust is crucial for an individual’s ongoing behavior when using information technology. This is also true in the internet-based medical service environment [85]. Studies have shown that the realization of internet-based medical services is divided into 2 phases: first, patients develop perceptions about the technical aspects of the health care platform, including its functionality, usefulness, and convenience [86]; second, patients evaluate the trustworthiness of the care provided and the service process [87]. Strong behavioral intentions only occur if older adults have a positive perception of the trustworthiness of the care.

Based on previous studies, this study further clarifies the influence of intergenerational support on older adults’ intention to adopt internet-based medical services. It constructs and validates an integrated model of the effect of health communication: intergenerational support → PEOU → PU → trust → intention to adopt internet-based medical services. As a new approach to medical care, internet-based medical services improve convenience and efficiency. With intergenerational support, older adults perceive the technical aspects (PEOU and PU) of these services more positively. This, in turn, enhances their trust and intention to use internet-based medical services, facilitating the integration of online care into health care.

Intergenerational support had a greater effect on the willingness of older adults to use internet-based medical services among those with high eHealth literacy. According to Jung et al [55], eHealth literacy enhances older adults’ ability to manage health-related issues effectively. Health literacy has also been positively associated with help-seeking intentions [88], older adults’ choice of physician, and their understanding of the physician’s recommendations [89]. Older adults with high eHealth literacy are thus more likely to have a higher intention to seek medical care on the internet when they receive support from their children and are more likely to participate in internet-based medical services actively for a better health care experience [90].

Previous studies have shown that PU and trust in online medical information [91] can improve eHealth literacy. However, this study demonstrated that the degree of intergenerational support for perceived technology, as well as trust in internet-based medical services, is deeper when older adults have higher eHealth literacy. According to Nie et al [92], people with higher eHealth literacy better assess the usefulness of searching for health information online. However, it may be that eHealth literacy increases older adults’ interest in health, their knowledge, and their expectations of health care [93] while inducing confidence and a sense of the efficacy of internet-based medical services.

Much of the health and medical information provided by internet-based medical service platforms originates from authoritative health care institutions and professionals, ensuring a high degree of accuracy. These platforms can support long-term disease management and health monitoring [94], allowing older people to access the health information they need. Bundorf et al [95] argued that this shift to searching for medical information online has obvious impacts on health behaviors as well as on treatment choices [96], improving older adults’ health beliefs and motivating them to engage in healthy activities [97]. There is also evidence that online medical information not only encourages self-initiated participation in health care [98] but also increases the likelihood that older adults will take an active role in disease management. It is thus reasonable to believe, based on the present results, that online medical information can play an active role in disease management and increase the willingness of older adults to use internet-based medical services.

### Limitations

Although this study initially verified the proposed theoretical model, certain limitations remain. First, the study focused exclusively on the PEOU and PU of internet-based medical services, leaving other related perceptions (eg, risks and benefits) unexplored. Moreover, it considered only the factors influencing older adults’ willingness to use internet-based medical services, without addressing motivation, sustained willingness to use, or actual usage. Finally, this study examined only the influence of the core variable of intergenerational support. Other factors, such as peer influence, social influence (eg, doctors’ recommendations), and subjective norms, could be explored in greater depth and incorporated into models in future research.

### Implications

Examining technology use among older adults is also a valuable starting point for addressing the range of social issues associated with aging [99]. This study’s focus on the needs of older adults regarding internet-based medical services, as well as their intentions to use such services, offers constructive insights for improving health equity and enhancing the inclusiveness and equity of internet-based medical services in particular [100]. Perceptions of and trust in technology are crucial factors in facilitating older adults’ adoption of internet-based medical services. Efforts to encourage older adults to use these services may first need to ensure that they recognize such services as not only useful and easy to use but also trustworthy.

Medical institutions and internet-based medical platforms can take the following measures: in platform design, they should fully consider the characteristics and needs of older adults, implement age-adapted designs, lower the barriers to use, and create an age-friendly online environment. Additionally, medical institutions and platforms must establish a highly credible environment by ensuring transparency in the treatment process and maintaining honesty and openness with patients. Protecting the personal information of older adults and preventing data breaches are essential to enhancing their confidence in using internet-based health care services.

Furthermore, the role of intergenerational support in enhancing older adults’ intention to seek medical care online must be emphasized. This highlights the importance of understanding the barriers older adults may face when using internet-based medical services, as well as the critical role children/young adults can play in facilitating the adoption of new technologies by older generations.

Within families, the younger generation should take the initiative to help older adults adapt to digital technology by offering emotional support and technical guidance while leveraging the advantages of the internet for health management.

The use of internet-based medical services by older adults holds significant value for management. First, it improves management efficiency. Internet-based medical services simplify traditional medical processes, enhancing hospital management efficiency. These platforms can allocate medical resources more effectively based on the health status and needs of older adults, optimizing resource utilization. Second, it fosters an innovative service model. Internet-based medical platforms can leverage technologies such as big data and artificial intelligence to deliver more precise medical services. Additionally, they can collaborate with other industries, such as care services for older adults, to innovate and expand service models. Third, it facilitates policy implementation. As a platform for policy dissemination, internet-based medical services can help raise awareness among older adults about national health and pension policies, thereby supporting the effective implementation of these policies. Fourth, it reflects social value. Internet-based medical services address the health needs of older adults, demonstrating social care and respect for older adults. This contributes to the transformation and upgrading of the national health care industry, ultimately improving the overall quality of medical services.

### Conclusions

Population aging is a long-term national issue in China, and the availability of medical services is directly linked to the quality of life for older adults. This study provides evidence that intergenerational support is a key factor in promoting the adoption of internet health care services by older adults. Encouraging children to offer guidance and assistance to their parents is essential. To increase older adults’ willingness to use internet health care, future efforts should focus not only on intergenerational support but also on improving the PEOU and usefulness of the technology, building trust, and fostering eHealth literacy among older adults.

## Appendix

Multimedia Appendix 1 Questionnaire.

Multimedia Appendix 2 Data (centralized processing).

Multimedia Appendix 3 Additional analysis.

## Abbreviations

AGFI: adjusted goodness of fit index
CFA: confirmatory factor analysis
CFI: comparative fit index
eHL: eHealth literacy
GFI: goodness of fit index
IFI: incremental fit index
KMO: Kaiser-Meier-Olkin
mHealth: mobile health
NNFI: nonnormed fit index
OMI: online medical information
PEOU: perceived ease of use
PU: perceived usefulness
RMSEA: root mean square error of approximation
SMCI: intention to seek medical care on the internet
