# Supplementary material for: Association Between Intergenerational Support, Technology Perception and Trust, and Intention to Seek Medical Care on the Internet Among Chinese Older Adults: Cross-Sectional Questionnaire Study
Source: J Med Internet Res. 2025 Jan 6;27:e65065. doi: 10.2196/65065 (PMC11747539; doi:10.2196/65065)
Supplement: Multimedia Appendix 1 [file jmir_v27i1e65065_app1.zip › a copy of the questionnaire.docx]

**Intergenerational Support and Older Adults' Intention**

**to Seek Medical Care on the Internet: Cross-Sectional Questionnaire Study**

Thank you very much for taking your valuable time to participate in this survey, which aims to understand intention of the older adults to seek medical treatment on the Internet.

**Below we have provided a picture of health care on the internet, please read what is in that picture and have a preliminary understanding of health care on the internet. The Investigators and researchers will choose to demonstrate to you the process of seeking health care on the Internet by giving you a face-to-face operation on how to seek health care on the Internet after you have read the pictures. Finally, please help us by completing the questions below.**

This survey is anonymous, there is no right or wrong in your answer, just choose according to the real idea. ***This survey will only be used for research data for research papers to ensure that all questions never involve your personal privacy.***

**Notes: for participants who choose to complete the questionnaire online, please click on the link to watch the “Healthcare on the Internet” video before answering the questions.**

https://v.douyin.com/iyjHXnpF/


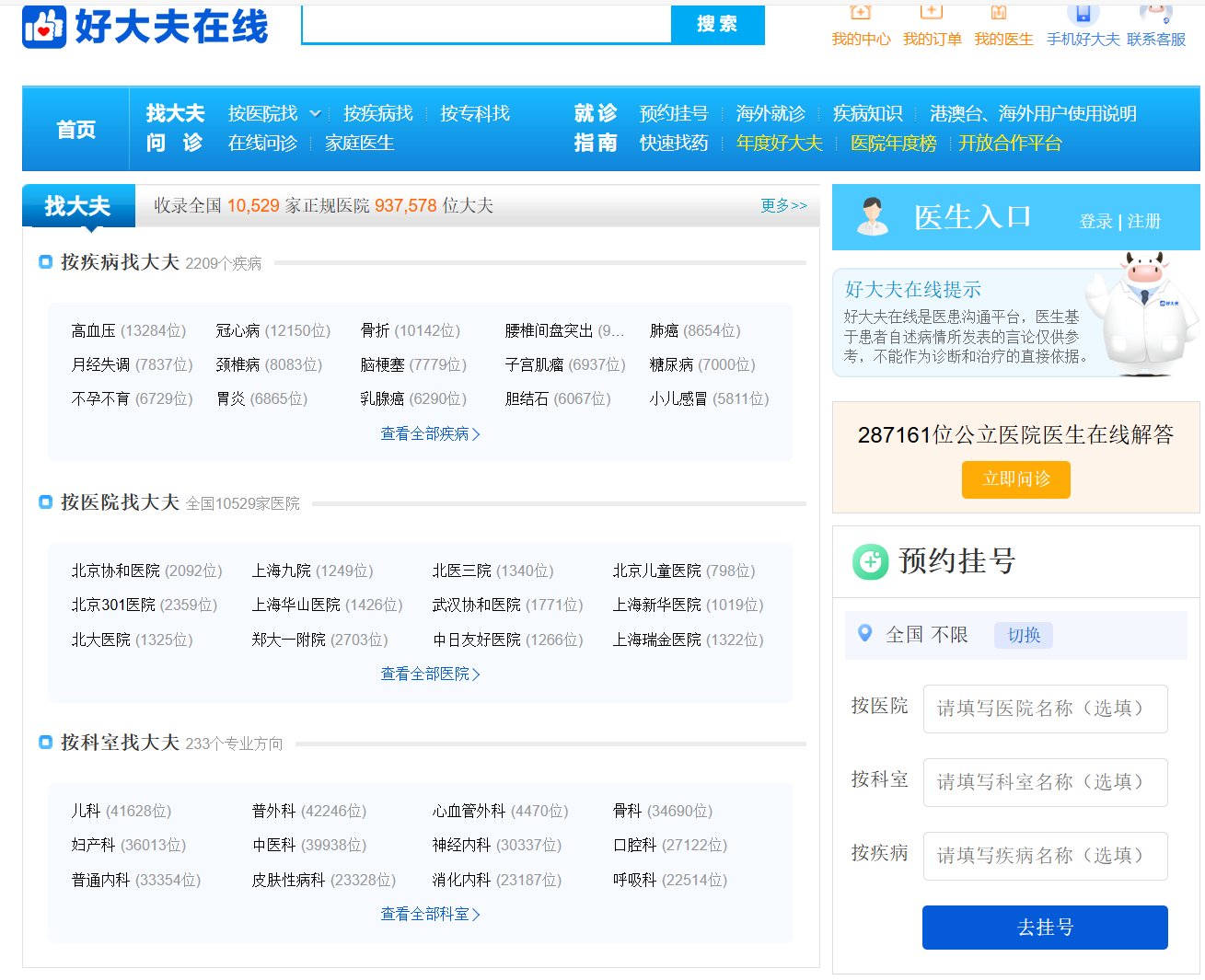


For questions 10 to 21, please put a tick in the box that you think is appropriate according to your actual situation, there is no right or wrong answer.

10. The following questions are about your children helping you seek medical care on the Internet.

|  | Strongly  disagree | disagree | Slightly disagree | Might consider | Slightly agree | agree | Strongly  agree |
| --- | --- | --- | --- | --- | --- | --- | --- |
| My children encourage me to seek medical care on the Internet, and I am willing to try. |  |  |  |  |  |  |  |
| My children guide I would be willing to try. |  |  |  |  |  |  |  |
| When I had problems with my internet health care, my children help me solve the problem, so I would be willing to try it. |  |  |  |  |  |  |  |

11. The following questions are about your Internet medical information exposure.

|  | Strongly  disagree | disagree | Slightly disagree | Might consider | Slightly agree | agree | Strongly  agree |
| --- | --- | --- | --- | --- | --- | --- | --- |
| I would search for medical information on the internet. |  |  |  |  |  |  |  |
| Online medical Information has taught me something about health. |  |  |  |  |  |  |  |
| I think online medical information is credible |  |  |  |  |  |  |  |
| I think online medical information is useful |  |  |  |  |  |  |  |

12. Please select Strongly Disagree: [OPTIONAL]*

A. strongly agree B. slightly agree C. strongly disagree

13. The following question items indicate your trust in Internet healthcare services.

|  | Strongly  disagree | disagree | Slightly disagree | Might consider | Slightly agree | agree | Strongly  agree |
| --- | --- | --- | --- | --- | --- | --- | --- |
| Most of the doctors on the online medical service platforms are health experts in their fields, and I have no doubt about their professionalism. |  |  |  |  |  |  |  |
| Generally, I trust health advice or tips from doctors on the internet. |  |  |  |  |  |  |  |
| Overall, I think the doctors on the Internet health care service platform are trustworthy |  |  |  |  |  |  |  |

14. The following question items indicate your perception of the usefulness of Internet medical services.

|  | Strongly  disagree | disagree | Slightly disagree | | Might consider | Slightly agree | agree | Strongly  agree |
| --- | --- | --- | --- | --- | --- | --- | --- | --- |
| I believe that using the internet for medical care can improve the efficiency of health care. |  |  | |  |  |  |  |  |
| I think it's better to communicate with doctors via the Internet than traditional offline visits |  |  | |  |  |  |  |  |
| Overall, I think online medical care is useful for health management. |  |  | |  |  |  |  |  |

15. The following question items indicate your perception of the ease of use of Internet healthcare services.

|  | Strongly  disagree | disagree | Slightly disagree | Might consider | Slightly agree | agree | Strongly  agree |
| --- | --- | --- | --- | --- | --- | --- | --- |
| I don't think it's difficult to use the Internet for health counseling |  |  |  |  |  |  |  |
| I think it's easy to learn how to use the internet to get medical care |  |  |  |  |  |  |  |
| I think the user interface of the Internet healthcare service platform is clear and easy to understand and operate |  |  |  |  |  |  |  |
| For sensitive health issues, I think online health counseling is easier than face-to-face counseling |  |  |  |  |  |  |  |
| Overall, I think internet healthcare is easy to use |  |  |  |  |  |  |  |

16. The following questions are about how you assess your level of eHealth literacy.

|  | Strongly  disagree | disagree | Slightly disagree | Might consider | Slightly agree | agree | Strongly  agree |
| --- | --- | --- | --- | --- | --- | --- | --- |
| I know how to find useful health resources and information online |  |  |  |  |  |  |  |
| I know how to use the health care type information I find on the internet to help myself. |  |  |  |  |  |  |  |
| I feel confident using the information I find online to make decisions related to my health |  |  |  |  |  |  |  |

17. The following questions indicate your intention to seek medical care on the Internet.

|  | Strongly  disagree | disagree | Slightly disagree | Might consider | Slightly agree | agree | Strongly  agree |
| --- | --- | --- | --- | --- | --- | --- | --- |
| When I'm face health problems, I think I'll solve them through internet health care. |  |  |  |  |  |  |  |
| I am willing to use internet health care services for health counseling, such as disease control. |  |  |  |  |  |  |  |
| I intend to use the internet frequently for medical care. |  |  |  |  |  |  |  |

1. Your age: ( )

A. 60-65 years old B. 66-70 years old C. 71-75 years old D. 76-80 years old E. 81 years old and above

2. Your gender: ( )

A. Male B. Female

3. Residence status: ( )

A. Living alone B. Living with spouse C. Living with children D. Living with extended family E. Others

4. Educational level: ( )

A. Not attending school B. Elementary school C. Junior high school D. High school/Junior high school

E. Bachelor's degree F. Graduate school and above

5. Occupation now or before retirement: ( )

A. public official/institution B. business manager/office general staff worker C. laborer

D. Farmer E. Self-employed F. Others

6. Currently your monthly income: (Unit: RMB/month) ( )

A.2000 and below B.2001 to 4000 C.4001 yuan to 6000

1. 6001 to 8000 E. More than 8000
2. 7. Where you live at present: ( )

A. Rural B. Urban

8. the number of your children: ( )

A.0 B.1 C.2 D.3 E.4 F.5 G.

E.4 F.5 G.6 or more

9. Your evaluation of your own health condition: ( )

A. very poor B. rather poor C. average D. rather good E. very good
