# Supplementary material for: Association Between Intergenerational Support, Technology Perception and Trust, and Intention to Seek Medical Care on the Internet Among Chinese Older Adults: Cross-Sectional Questionnaire Study
Source: J Med Internet Res. 2025 Jan 6;27:e65065. doi: 10.2196/65065 (PMC11747539; doi:10.2196/65065)
Supplement: Multimedia Appendix 3 [file jmir_v27i1e65065_app3.zip › Supplementary Materials.doc]

Table S1. Measurement items.

| variable | Content |
| --- | --- |
| Intergenerational Support | (IS1) My children encourage me to seek medical care on the internet, and I am willing to try. |
|  | (IS2) My children guide I would be willing to try. |
|  | (IS3) When I had problems with my internet health care, my children help me solve the problem, so I would be willing to try it. |
| Trust | (T1) Most of the doctors on the online medical service platforms are health experts in their fields, and I have no doubt about their professionalism. |
|  | (T2) Generally, I trust health advice or tips from doctors on the internet. |
|  | (T3) Overall, I think the doctors on the internet medical service platform are trustworthy. |
| Perception Usefulness | (PU1) I believe that using the internet for medical care can improve the efficiency of health care. |
|  | (PU2) I think it's better to communicate with doctors via the internet than traditional offline visits. |
|  | (PU3) Overall, I think online medical care is useful for health management. |
| Perception Ease of Use | (PEOU1) I don't think it's difficult to use the internet for health counseling. |
|  | (PEOU2) I think it's easy to learn how to use the internet to get medical care. |
|  | (PEOU3) I think the user interface of the internet health care service platform is clear and easy to understand and operate. |
|  | (PEOU4) For sensitive health issues, I think online health counseling is easier than face-to-face counseling. |
|  | (PEOU5) In general, I think internet health care is easy to use. |
| E-Health Literacy | (eHL1) I know how to find useful health resources and messages online. |
|  | (eHL2) I know how to use the health care-type information I find on the internet to help myself. |
|  | (eHL3) I feel confident using the information I find online to make decisions related to my health. |
| Online Medical Information | (OMI1) I would search for medical information on the internet. |
|  | (OMI2) Online medical Information has taught me something about health. |
|  | (OMI3) I think online medical information is credible. |
|  | (OMI4) I think online medical information is useful. |
| Intention to Seek Medical Care on Internet | (SMCI 1) When I'm face health problems, I think I'll solve them through internet health care. |
|  | (SMCI 2) I am willing to use internet health care services for health counseling, such as disease control. |
|  | (SMCI 3) I intend to use the internet frequently for medical care. |

Table S2. Measurement model.

| Goodness-of-fit indices | Recommend Value | Value Obtained |
| --- | --- | --- |
| X²/df | <5 acceptable; <3 desirable | 2.273 |
| GFI | >0.8 acceptable; >0.9 desirable | 0.958 |
| AGFI | >0.8 acceptable; >0.9 desirable | 0.945 |
| IFI | ＞0.9 | 0.980 |
| CFI | ＞0.9 | 0.980 |
| NNFI (TLI) | ＞0.9 | 0.976 |
| RMSEA | <0.08 | 0.036 |

Table S3. Confirmatory factor analysis

| Variables | Items | Factor loadings | CR | AVE |
| --- | --- | --- | --- | --- |
| Intergenerational Support | IS1 | 0.854 | 0.887 | 0.723 |
|  | IS2 | 0.841 |  |  |
|  | IS3 | 0.856 |  |  |
| Online Medical Information | OMI1 | 0.789 | 0.896 | 0.684 |
|  | OMI2 | 0.872 |  |  |
|  | OMI3 | 0.818 |  |  |
|  | OMI4 | 0.826 |  |  |
| Trust | T1 | 0.794 | 0.869 | 0.690 |
|  | T2 | 0.814 |  |  |
|  | T3 | 0.881 |  |  |
| Perception Usefulness | PU1 | 0.845 | 0.888 | 0.726 |
|  | PU2 | 0.835 |  |  |
|  | PU3 | 0.876 |  |  |
| Perceived Ease of Use | PEOU1 | 0.835 | 0.906 | 0.659 |
|  | PEOU2 | 0.809 |  |  |
|  | PEOU3 | 0.791 |  |  |
|  | PEOU4 | 0.762 |  |  |
|  | PEOU5 | 0.858 |  |  |
| E-Health Literacy | eHL1 | 0.861 | 0.894 | 0.739 |
|  | eHL2 | 0.865 |  |  |
|  | eHL3 | 0.852 |  |  |
| Intention to Seek Medical Care on Internet | SMCI 1 | 0.868 | 0.896 | 0.742 |
|  | SMCI 2 | 0.872 |  |  |
|  | SMCI 3 | 0.843 |  |  |

Table S4. Discriminant validity

|  | IS | OMI | T | PU | PEOU | eHL | SMCI |
| --- | --- | --- | --- | --- | --- | --- | --- |
| IS | **0.85** |  |  |  |  |  |  |
| OMI | 0.115 | **0.827** |  |  |  |  |  |
| T | 0.439 | 0.261 | **0.831** |  |  |  |  |
| PU | 0.381 | 0.17 | 0.415 | **0.852** |  |  |  |
| PEOU | 0.254 | 0.219 | 0.367 | 0.252 | **0.812** |  |  |
| eHL | 0.143 | 0.144 | 0.232 | 0.214 | 0.13 | **0.859** |  |
| SMCI | 0.507 | 0.233 | 0.469 | 0.433 | 0.325 | 0.222 | **0.861** |

Table S5. Measurement model.

| Goodness-of-fit indices | Recommend Value | Value Obtained |
| --- | --- | --- |
| X²/df | <5 acceptable; <3 desirable | 2.784 |
| GFI | >0.8 acceptable; >0.9 desirable | 0.954 |
| AGFI | >0.8 acceptable; >0.9 desirable | 0.94 |
| IFI | ＞0.9 | 0.975 |
| CFI | ＞0.9 | 0.975 |
| NNFI (TLI) | ＞0.9 | 0.971 |
| RMSEA | <0.08 | 0.043 |
